# Terahertz-Rate Kerr-Microresonator Optical Clockwork

**DOI:** 10.1103/physrevx.9.031023

**Published:** 2019

**Authors:** Tara E. Drake, Travis C. Briles, Jordan R. Stone, Daryl T. Spencer, David R. Carlson, Daniel D. Hickstein, Qing Li, Daron Westly, Kartik Srinivasan, Scott A. Diddams, Scott B. Papp

**Affiliations:** 1 Time and Frequency Division, National Institute of Standards and Technology, 385 Broadway, Boulder, Colorado 80305, USA; 2 Department of Physics, University of Colorado, Boulder, Colorado, 80309, USA; 3 Center for Nanoscale Science and Technology, National Institute of Standards and Technology, Gaithersburg, Maryland 20899, USA

## Abstract

Kerr microresonators generate interesting and useful fundamental states of electromagnetic radiation through nonlinear interactions of continuous-wave (CW) laser light. With photonic-integration techniques, functional devices with low noise, small size, low-power consumption, scalable fabrication, and heterogeneous combinations of photonics and electronics can be realized. Kerr solitons, which stably circulate in a Kerr microresonator, have emerged as a source of coherent, ultrafast pulse trains and ultra-broadband optical-frequency combs. Using the f-2f technique, Kerr combs can support carrier-envelope-offset phase stabilization to enable optical synthesis and metrology. Here, we introduce a Kerr-microresonator optical clockwork, which is a foundational device that distributes optical-clock signals to the mode-difference frequency of a comb. Our clockwork is based on a silicon-nitride (Si_3_N_4_) microresonator that generates a Kerr-soliton frequency comb with a repetition frequency of 1 THz. We measure our terahertz clockwork by electro-optic modulation with a microwave signal, enabling optical-based timing experiments in this wideband and high-speed frequency range. Moreover, by EO phase modulation of our entire Kerr-soliton comb, we arbitrarily generate additional CW modes between the 1-THz modes to reduce the repetition frequency and increase the resolution of the comb. Our experiments characterize the absolute frequency noise of this Kerr-microresonator clockwork to one part in 10^17^, which is the highest accuracy and precision ever reported with this technology and opens the possibility of measuring high-performance optical clocks with Kerr combs.

## I. INTRODUCTION

Optical-atomic clocks [1], which are among the most precise metrological instruments currently available, provide continuous-wave (CW) laser radiation that is stabilized to a narrow-linewidth atomic transition. Optical-frequency combs provide a clockwork to coherently transfer the stability of an optical clock to all the comb modes and to a microwave signal derived from the comb repetition frequency. The latter capability, called optical-frequency division (OFD), was developed for optical timekeeping [2,3], and it leverages the frequency multiplication inherent in the comb’s mode spectrum [4], namely, $\nu_{n}={ƒ}_{\text{ceo}}+nf_{\text{rep}}$, where $f_{\text{ceo}}$ is the carrier-envelope offset frequency and $f_{\text{rep}}$ is the repetition frequency. To implement OFD, mode n of an optical frequency comb is phase locked to an optical reference such that $f_{\text{rep}}=(\nu_{n}-f_{\text{ceo}})/n$, hence dividing the optical clock carrier frequency to a lower frequency by a factor of n and concurrently reducing the phase noise by approximately a factor $20\;{\text{log}}_{10}(n)$. High-performance tabletop oscillators make use of this property [5–11].

Dissipative-Kerr-soliton generation in Kerr-microresonators [12] is a recent advance that enables mode-locked frequency combs in miniature devices with relatively low power consumption and the potential for planar integration. This work builds on decades of knowledge in soliton nonlinear optics [13]. The development of Kerr-soliton microresonator frequency combs (“microcombs” in this paper) has led to explorations of novel nonlinear states of light [14,15], microresonator solitons [16,17], and demonstrations of functional devices such as clocks [18], optical synthesizers [19,20], dual-comb spectrometers [21,22], massively parallel communications [23], and high-speed ranging [24]. Recent experiments have developed access to the carrier-envelope offset frequency of microcombs, $f_{\text{ceo}}=\nu_{p}-Nf_{\text{rep}}$, where $\nu_{p}$ is the pump laser frequency and N is the comb mode number to measure $f_{\text{ceo}}<f_{\text{rep}}$, through 2f-3f [25,26] and f-2f measurements [19,20].

Developing an integrated-photonics clockwork will enable new applications of optical timekeeping. The recently developed very high $f_{\text{rep}}\approx 0.2–1$ THz soliton combs [16,27] that leverage careful engineering of the waveguide dimensions for spectral bandwidth and $f_{\text{ceo}}$ control [19] are useful for microwave, mm-wave, and THz photonic-microwave generation [28]. Phase stabilization of $f_{\text{ceo}}$ is unavoidably required since real time tracking and correction for the comb repetition frequency—the output of the clockwork whose stability ought to exceed existing microwave oscillators—are not possible.

Here, we report a Kerr-microresonator clockwork that coherently divides the frequency of an optical-clock laser to a terahertz signal. Our experiments use an f-2f self-referenced Si_3_N_4_ microcomb and $f_{\text{ceo}}$ phase stabilization, and with these tools, we show how to perfectly divide and transfer the frequency stability of the clock laser to $f_{\text{rep}}$ of the Si_3_N_4_ microcomb. We verify the fractional-frequency accuracy and precision of our Kerr-microresonator clockwork to the 10^−17^ level after continuous, glitch-free operation for two hours. This verification experiment is enabled by terahertz-rate frequency metrology with respect to a separate clockwork system, which is based on a f-2f self-referenced electro-optic (EO) modulation comb (here-after referred to as the reference comb) [29]. Comparison of these two ultrahigh-speed clockworks jointly evaluates their residual stability, including the optical and microwave network that interfaces them. Moreover, by performing our experiments at terahertz rates, frequency multiplication offers exceptional precision with respect to microwave oscillators. A further benefit of combining microcomb and EO-comb technology is that direct EO modulation of our Si_3_N_4_ comb effectively reduces its mode spacing and increases its resolution to an electronically detectable rational submultiple of 1 THz. With this technique, we demonstrate 33-GHz or 8-GHz mode spacing combs with up to 43 THz of optical bandwidth. Our work both explores a new range of accuracy and precision with Kerr-soliton comb technology and highlights the advantages of terahertz-rate measurements.

## II. KERR-MICRORESONATOR CLOCKWORK

**FIG. 1.**
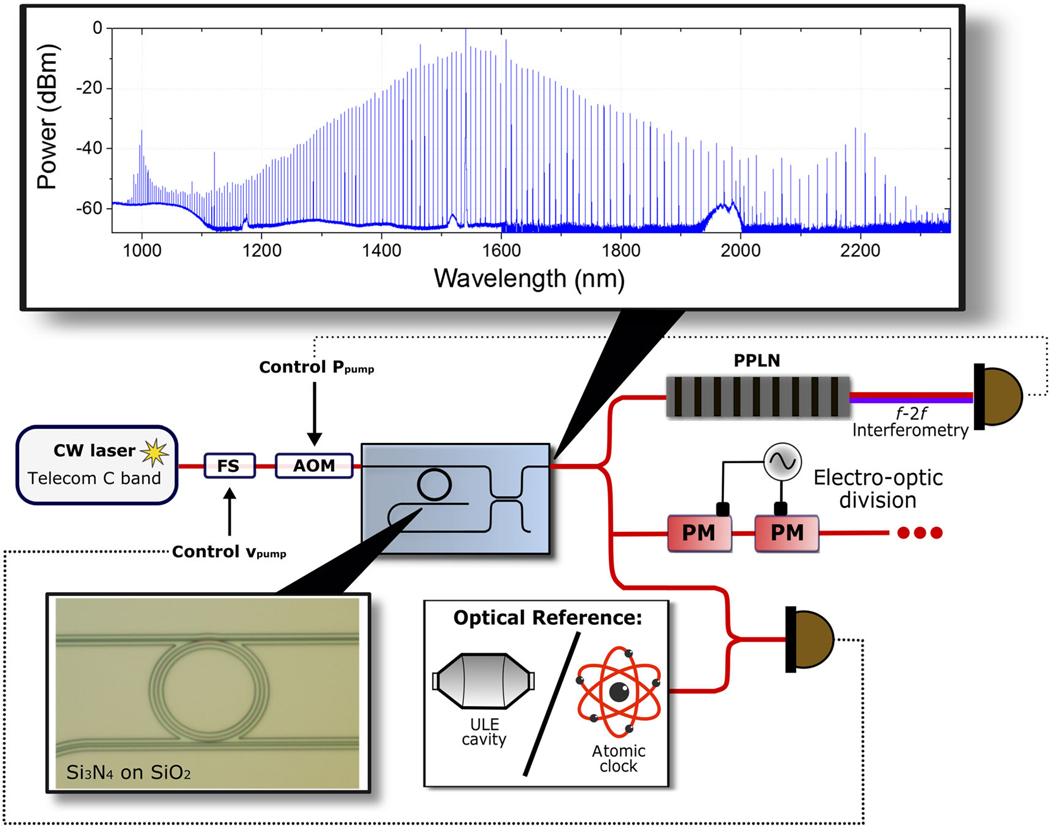
Schematic of the Kerr-microresonator clockwork. The microresonator is a Si_3_N_4_ ring with radius of 23 *μ*m. It is pumped with a CW laser that is coupled into a 720-nm-wide access waveguide (upper waveguide in this picture) via lensed fibers. The resultant comb spans more than an octave, and wavelengths from 900 to 1900 nm are outcoupled from the resonator via the access waveguide through port, while wavelengths greater than 1900 nm are outcoupled via a drop port to a 1200-nm-wide waveguide (lower waveguide in this picture). The comb light is recombined via an adiabatic dichroic coupler before leaving the chip. The microcomb is stabilized through f-2f interferometry and locking to an optical-frequency reference. The f-2f technique is carried out with a waveguide PPLN (periodically poled lithium niobite) and a PID (proportional-integral-derivative) feedback circuit controlling the CW pump intensity via an acousto-optic modulator (AOM). The optical reference (a ULE-cavity-stabilized laser in this paper) creates a heterodyne beat with one of the comb modes, and a second PID feedback circuit controls the CW pump frequency via a QPSK modulator (FS). When both PID feedback circuits are operational, the fractional stability of the optical cavity is transferred to $f_{\text{rep}}$, using OFD. The full comb spectrum is also sent through a series of electro-optic phase modulators (PM) to reduce the effective repetition frequency.

Figure 1 shows our Kerr-microresonator clockwork setup. The microresonator is a silicon-nitride (Si_3_N_4_ here-after truncated to SiN) microring, fabricated by way of low-pressure chemical-vapor deposition (LPCVD), electron-beam lithography, and chemical etching [16]. The device has an unloaded mode quality factor of approximately 2.5 × 10^6^ and a radius of approximately 23 *μ*m. The SiN waveguide is air clad on three sides, and its width, height, and radius are carefully chosen for the desired dispersion and carrier-envelope-offset frequency [16,19]. The resonator is pumped with approximately 200 mW of chip-coupled CW laser power from a 1540-nm external-cavity diode laser (ECDL); the frequency and power of the pump laser are controlled using a frequency shifter (FS) and an acousto-optic modulator (AOM), respectively. The FS enables laser-frequency sweeps across a SiN ring resonance that are faster than the thermal heating rate of the device, thereby avoiding a large thermal bistability and making soliton formation rather straightforward; see Refs. [30] and [19] for more details. The dispersion of the SiN resonator enables generation of a soliton microcomb with modes exceeding 1 nW (often 10 nW) of optical power per mode from 960 nm to 2300 nm, and the power at either extreme is increased by dispersive waves [19]; see Fig. 1.

The general principle of clockwork operation follows the equation $f_{\text{rep}}=(\nu_{p}-f_{\text{ceo}})/N$. Operationally, we phase lock one of the microcomb’s modes to an optical-frequency reference and simultaneously stabilize $f_{\text{ceo}}$. In this paper, the optical-frequency reference is provided by a CW laser that is locked via the Pound-Drever-Hall method to a single mode of an ultra-low-expansion (ULE) cavity [31]. This cavity-stabilized laser achieves a fractional-frequency stability of approximately 10^−15^ for 1-s measurements, and it has a typical drift rate of 100 mHz/s. In particular, the central microcomb mode, corresponding to the pump laser, is phase locked to the cavity-stabilized laser via an electronic feedback circuit to the FS. The choice of optical reference is flexible; another possibility is to use a compact atomic reference based on the two-photon Rb transition at 778 nm [32].

We phase lock $f_{\text{ceo}}$, obtained using the f-2f technique, to a hydrogen-maser-referenced microwave oscillator, using the pump-laser intensity. This results in the phase-coherent conversion of the optical reference to $f_{\text{rep}}$ of the microcomb. Since full integration of the SiN microresonator and PPLN waveguide on the same chip has not yet been achieved, the doubling process must be done after outcoupling light from the chip with the SiN resonator and recoupling onto the chip with the PPLN waveguide. The loss associated with these processes (approximately 13 dB total), the efficiency of the PPLN (approximately 30% per W on chip), and the scaling of the doubling process with the square of the intensity of the 1998-nm light all conspire to make direct doubling of a 1998-nm comb mode infeasible. Instead, we send an auxiliary laser (Newport TLB-6736 ECDL, wavelength of 1998 nm) through the PPLN waveguide to collect 10 mW of 1998-nm light and 0.03 mW of 999-nm light. Two optical heterodynes are formed with the comb modes at 1998 nm and 999 nm, and these two frequencies are coherently mixed together in real time with a 2∶1 ratio to directly detect $f_{\text{ceo}}$. Therefore, our $f_{\text{ceo}}$ signal has no contribution from the auxiliary laser and is independent of the auxiliary laser frequency. Moreover, because of this coherent mixing, the auxiliary laser frequency and its noise do not contribute to the terahertz-rate output of our clockwork; the measurements of $f_{\text{ceo}}$ and the clockwork output that we present in the following section demonstrate this feature.

## III. CHARACTERIZING THE KERR-MICRORESONATOR CLOCKWORK

We characterize the residual-noise contribution of the Kerr-microresonator clockwork to its optical-frequency reference. We perform these measurements by comparing the result of OFD—the 1-THz repetition frequency of the microcomb—to the reference comb that also implements OFD of the same ULE cavity-stabilized laser. In this frequency comparison of two 1-THz signals, the residual noise we measure indicates the contribution of both OFD systems and the microwave and optical network that connects them. Our reference is an EO frequency comb with 10-GHz repetition frequency that is stabilized by super-continuum generation with a SiN photonic-chip waveguide and f-2f interferometry. Reference [29] provides a detailed functional explanation of the reference EO comb.

**FIG. 2.**
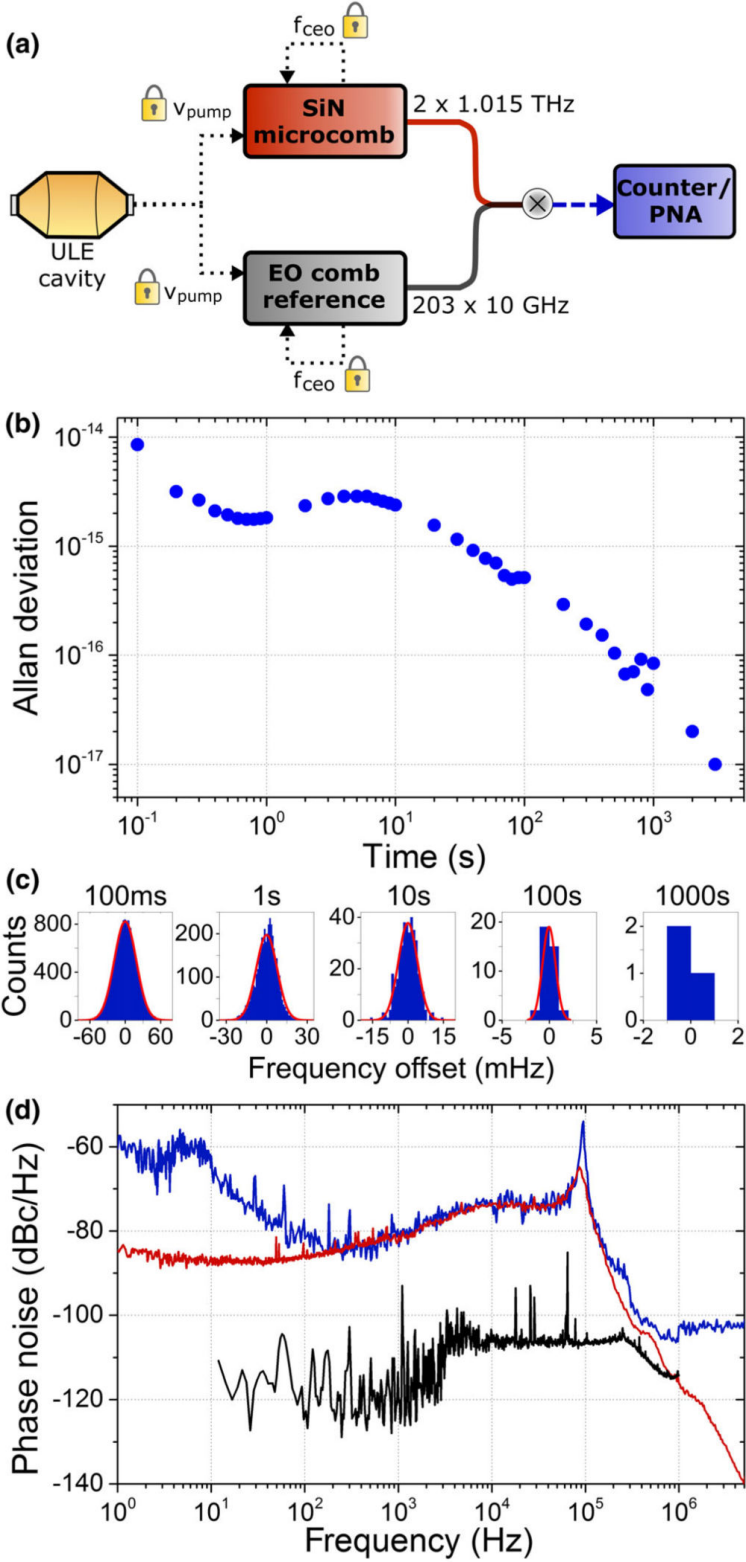
Studying the residual noise of the Kerr-microresonator clockwork. (a) The principle of our measurements is parallel operation of two clockworks, the microcomb and the EO reference comb. We measure and subtract optical-heterodyne beats between the combs at 1540 nm and 1524 nm, which characterizes the precise difference in the combs’ repetition frequencies $f_{\text{diff}}=2*f_{\text{rep SiN}}-203*f_{\text{rep EO}}$. (b) Allan deviation of $f_{\text{diff}}$. (c) Histograms of a 2-h measurement of $f_{\text{diff}}$ relating the absolute frequency accuracy of the two clockworks by direct comparison to its expected value. (d) Phase-noise spectrum of $f_{\text{diff}}$ (blue), $f_{\text{ceo}}$ (red), and the reference comb $f_{\text{rep EO}}$ (black). The reference comb contribution is shown to be insignificant.

Figure 2(a) shows the concept of the residual-noise measurement. The microcomb and the reference comb operate in parallel, and to compare them, we measure the 203rd harmonic of the reference-comb repetition frequency with respect to the second harmonic of the microcomb repetition frequency. We obtain this comparison of the two combs directly, without the need to detect any signals at 2 THz, by forming optical-heterodyne beats of the reference comb and microcomb modes at 1540 nm and 1524 nm, respectively. The difference of these heterodyne signals, which we obtain by electronic frequency mixing, directly reflects the difference of the two clockworks; we analyze this signal with a frequency counter and a phase-noise analyzer. This procedure highlights the advantage of a terahertz-rate clockwork, namely, that electronic noise from frequency mixing, amplification, and digitization of baseband signals is straightforwardly much lower than the clockwork signal itself.

In practice, the two comb systems exist in separate labs at NIST, and the reference comb is delivered to the microcomb lab through about 200 meters of optical fiber. We use an optical phase lock of the microcomb pump laser to one mode of the reference comb. This configuration allows for an almost perfect cancellation of the LE cavity laser’s frequency drift; see Supplemental Material [33] for more information on how we process our data. The result of our residual-noise measurement is a frequency-counter record and phase-noise analysis of $f_{\text{diff}}=2*f_{\text{rep SiN}}-203*f_{\text{rep EO}}$, which are shown in Figs. 2(b)–(d) and present a detailed picture of our clockwork’s performance. Here, $f_{\text{diff}}$ is a quantity that we can predict based on the frequency set points of our phase-locked combs. Moreover, the inherently terahertz-frequency contributions of $f_{\text{diff}}$ explain its resilience against baseband contributions from amplifiers, mixers, and analyzers. The Supplemental Material [33] contains more information regarding the measurement setup.

We verify that the two clockworks are accurate by subtracting our measurements of $f_{\text{diff}}$ from its expected value; see Fig. 2(c). A zero-frequency mean of the histograms in Fig. 2(c), within an uncertainty indicated by the Allan deviation presented in Fig. 2(b), indicates an accurate clockwork. Since the two combs share a common optical clock reference, $f_{\text{diff}}$ of their divided outputs can, in principle, be independent of drifts in the common optical clock frequency. However, since the division ratios, N=192 for the SiN comb and N=19 339 for the EO comb, are not commensurate and cannot be straightforwardly set this way in our current experiment [29], $f_{\text{diff}}$ drifts in time at less than 1.5 *μ*Hz/s according to the absolute optical-frequency drift of the ULE-cavity-stabilized laser. We track this drift independently and synchronously by frequency counting $f_{\text{rep EO}}$ with respect to a hydrogen-maser reference; see Supplemental Material [33] for more details. Without any assumption beyond the integer N values of the clockworks, we correct the measured $f_{\text{diff}}$. The data are presented as histograms with different timing bins in Fig. 2(c), and the mean error in $f_{\text{diff}}$ from the measured $f_{\text{repEO}}$ value (zero on the Fig. 2(c) *x* axis) is −25 *μ*Hz ± 95 *μ*Hz.

Our measurements capture the residual noise of the microcomb clockwork, as described above. Specifically, Fig. 2(b) indicates the Allan deviation of the two parallel clockworks with the measured cavity drift removed. From this measurement, we show that the residual noise of the microcomb clockwork is 1 part in 10^15^ for 1-s measurement durations and reduces to 1 part in 10^17^ after 2 h of measurement [Fig. 2(b)], which, to our knowledge, is the initial demonstration of complete optical frequency division with a microcomb and is a factor of 30 improvement over the most precise microcomb measurements reported to date [19]. Presumably, the Allan deviation performance plateau for 1–10-s measurements contains contributions from environmental fluctuations in the optical network that connects the two OFD systems since we did not actively stabilize path lengths. Importantly, this measurement of 1 part in 10^17^ represents a fundamental test of the intrinsic accuracy of the THz clockwork (i.e., the microcomb ability to accurately divide an optical clock), independent of the details of the optical clock.

We also present the phase-noise spectrum of $f_{\text{diff}}$ [Fig. 2(d)], which provides detailed insight into the performance of our clockwork. Three traces characterize this data, including the negligible contribution of the reference comb (black trace), the noise of $f_{\text{diff}}$ itself (blue trace), and the noise of $f_{\text{ceo}}$ (red trace) from which we can also infer the phase-noise spectrum of $f_{\text{diff}}$. Note that we obtain these spectra while the combs are phase locked, as described above; thus, they indicate a residual-phase-noise contribution within our servo bandwidth of approximately 100 kHz. In our current configuration, the repetition-frequency noise is dominant, indicated by the agreement of the $f_{\text{rep}}$ (seen on $f_{\text{diff}}$) and $f_{\text{ceo}}$ data when $f_{\text{rep}}$ is scaled by the appropriate factor of N=192. Therefore, the residual phase-noise contribution of the microcomb pump-laser phase locking is relatively low. Understanding why $f_{\text{rep}}$ is the dominant contribution is an important direction for future microcomb experiments and applications [34]. Nevertheless, these data highlight the strong correlation of $f_{\text{rep}}$ and $f_{\text{ceo}}$, indicating the capability for precision optical, terahertz, and microwave metrology in a single integrated-photonics device and mitigation of technical noise sources of our system such as the auxiliary laser.

## IV. MICROCOMB REPETITION-RATE REDUCTION FROM 1 THZ

The benefits of our microcomb system are an octave-spanning output spectrum, relatively high output power per mode at relatively low pump power, and photonic-chip integration. However, the 1-THz repetition frequency is challenging to measure with a photodetector and microwave electronics. Additionally, such a terahertz microcomb is not ideal for some applications because the broad overall spectral coverage is relatively undersampled. Here, we show how to address these shortcomings through the use of EO phase modulators [35], which effectively reduce the 1-THz repetition frequency to an, in principle, arbitrarily low value. This method is a convenient and efficient technique to build a user-defined frequency comb from our base octave-span microcomb.

**FIG. 3.**
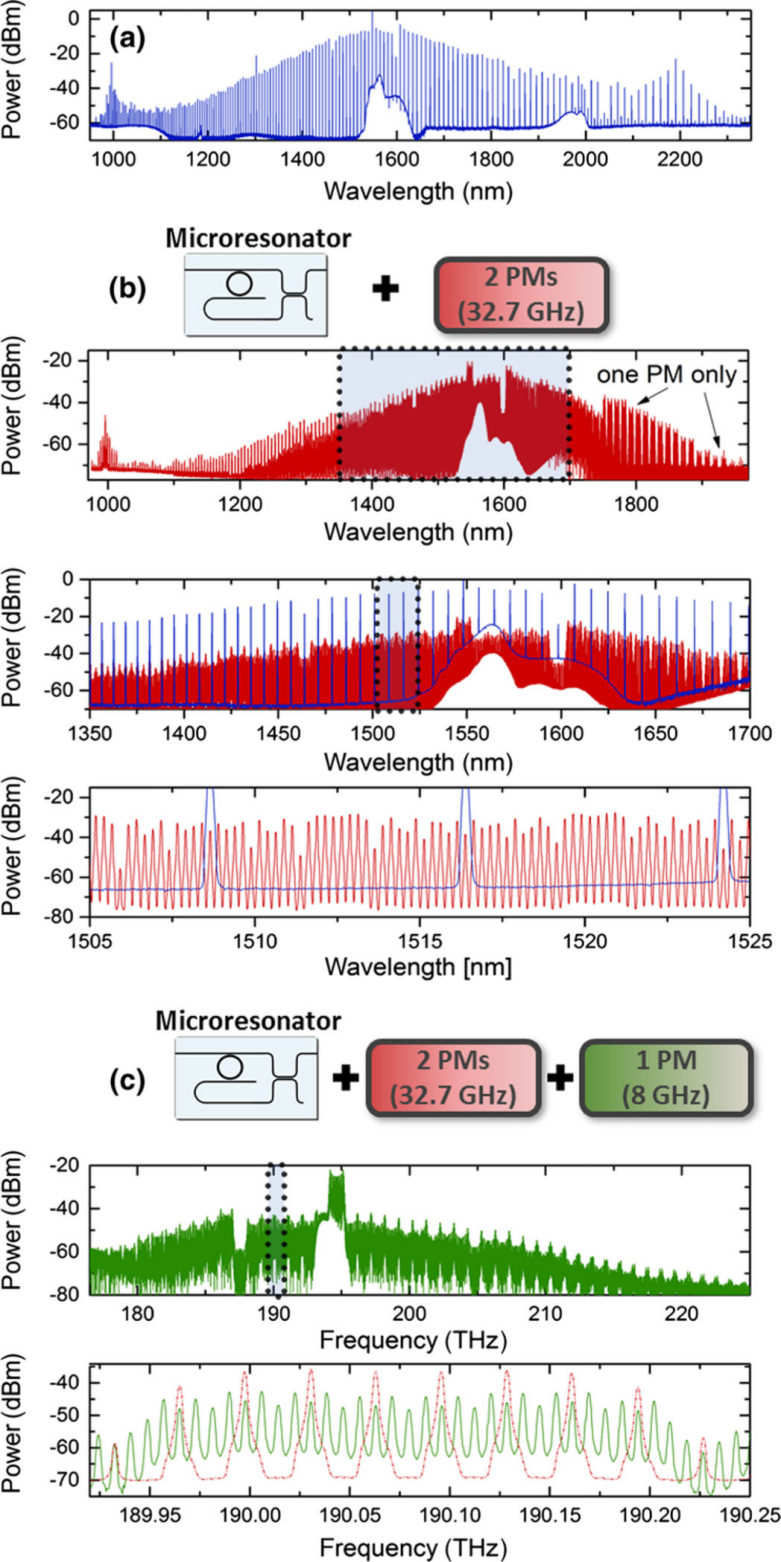
Reduction of the 1-THz SiN microcomb mode spacing through EO phase modulation. (a) Microcomb spectrum coupled out of the SiN waveguide, using lensed fibers. (b) The full microcomb spectrum (blue) is sent through two low-$V_{\pi }$ electro-optic phase modulators (PM), which are driven at roughly 32.7 GHz ($f_{\text{rep SiN}}/31$). These modulators are optimized for operation at 1550 nm; however, they perform well over a relatively large bandwidth, and the resulting spectrum includes a filled-in 32.7-GHz comb that spans nearly 350 nm (red). (c) Demonstrating further reduction, we send the 32.7-GHz comb (red) through an additional PM driven at 8 GHz. The mode spacing of the resulting spectrum (green) has been divided by 124 from the original microcomb $f_{\text{rep}}$. (This spectrum is shown in frequency units in contrast to the previous panels, shown in wavelength.)

Figure 3 shows the results of reducing the terahertz repetition frequency. The EO modulators generate nearly arbitrarily spaced interleaving combs about each SiN microcomb mode. Figure 3(a) shows the full spectrum outcoupled from the microresonator and output from the photonic chip, a fraction of which is sent through two low-$V_{\pi }$ phase modulators [Fig. 3(b)]. By driving these modulators at roughly $f_{\text{rep}}/31$ [in Fig. 3(b), $f_{\text{EO}}=32.6749009\;\text{GHz}$], we generate EO subcombs that are nearly subharmonic of the microcomb $f_{\text{rep SiN}}$; the difference between $f_{\text{rep SiN}}$ and $f_{\text{EO}}$ yields a low-frequency (about 10 MHz) heterodyne beat note between adjacent subcombs. In the case of OFD, the modulators facilitate readout of the clockwork. In past explorations, electro-optic phase modulation has been used in a feedback loop to stabilize $f_{\text{rep SiN}}$ directly [35]. In that method of operation, the stability of the RF reference driving the modulators is transferred to the stability of each microcomb mode. Conversely, in the OFD configuration, an electronic phase lock of the RF drive can be used to optimally stabilize the EO subcombs to the superior stability of the terahertz microcomb mode spacing. This straightforward setup would require a tunable RF reference to generate $f_{\text{EO}}$, digital division of this signal by the number of EO sidebands between microcomb modes (31, in this case), and finally using the divided RF to stabilize the heterodyne beat between adjacent subcombs through electronic feedback to the tunable RF reference. Such a setup would remove the noise associated with the generation of EO subcombs and allow for clock readout on $f_{\text{EO}}$.

Additional modulators can be included [Fig. 3(c)] to further subdivide the mode spacing. These modulators provide extra tunability of the optical mode frequencies without sacrificing the microcomb $f_{\text{rep}}$ readout capability. In Figs. 3(b) and 3(c), the shaded regions on the upper panels correspond to the enlarged spectrum in the panel below. Phase modulation of the spectra in Fig. 3(a) results in a more finely spaced but still optically broad spectrum of 43 THz (325 nm) at 32.5-GHz spacing and 35 THz (270 nm) at 8-GHz spacing. This capability, which could be straightforwardly refined for user-defined applications beyond the demonstrations reported here, is a powerful approach for experiments ranging from optical communications, dual-comb measurements, calibration of astronomical spectrographs, sensitive optical detection of signals, and various other directions.

## V. SUMMARY

In conclusion, this work demonstrates the utility and generality of Kerr-microresonator frequency combs in a laboratory setting, as well as paths for further development. We present a terahertz clockwork with 0.01-mHz accuracy and precision, opening a new measurement regime with Kerr combs. Terahertz frequency metrology can offer unique advantages both in terms of resilience against electronic-noise contributions, as we have shown here, and opportunities to bootstrap microwave frequencies from the uniquely high-frequency $f_{\text{ceo}}$ settings available in microcombs. Additionally, we demonstrate flexible repetition-frequency reduction from the native terahertz of our microcomb. This may be enabling for future avenues of research, from the many applications of spectroscopy, to further frequency-comb development like a fully integrated-photonics clockwork, to massively parallel telecommunication.
